# Postembryonic lineages of the *Drosophila* ventral nervous system: Neuroglian expression reveals the adult hemilineage associated fiber tracts in the adult thoracic neuromeres

**DOI:** 10.1002/cne.23988

**Published:** 2016-03-09

**Authors:** David Shepherd, Robin Harris, Darren W. Williams, James W. Truman

**Affiliations:** ^1^ School of Biological SciencesBangor UniversityBangorGwyneddUK; ^2^ HHMI‐Janelia Research CampusAshburnVirginiaUSA; ^3^ MRC Centre for Developmental NeurobiologyKing's College LondonLondonUK

**Keywords:** neurogenesis, metamorphosis, neuroblast, development

## Abstract

During larval life most of the thoracic neuroblasts (NBs) in *Drosophila* undergo a second phase of neurogenesis to generate adult‐specific neurons that remain in an immature, developmentally stalled state until pupation. Using a combination of MARCM and immunostaining with a neurotactin antibody, Truman et al. (2004; Development 131:5167–5184) identified 24 adult‐specific NB lineages within each thoracic hemineuromere of the larval ventral nervous system (VNS), but because of the neurotactin labeling of lineage tracts disappearing early in metamorphosis, they were unable extend the identification of these lineages into the adult. Here we show that immunostaining with an antibody against the cell adhesion molecule neuroglian reveals the same larval secondary lineage projections through metamorphosis and bfy identifying each neuroglian‐positive tract at selected stages we have traced the larval hemilineage tracts for all three thoracic neuromeres through metamorphosis into the adult. To validate tract identifications we used the genetic toolkit developed by Harris et al. (2015; Elife 4) to preserve hemilineage‐specific GAL4 expression patterns from larval into the adult stage. The immortalized expression proved a powerful confirmation of the analysis of the neuroglian scaffold. This work has enabled us to directly link the secondary, larval NB lineages to their adult counterparts. The data provide an anatomical framework that 1) makes it possible to assign most neurons to their parent lineage and 2) allows more precise definitions of the neuronal organization of the adult VNS based in developmental units/rules. J. Comp. Neurol. 524:2677–2695, 2016. © 2016 The Authors The Journal of Comparative Neurology Published by Wiley Periodicals, Inc.

Understanding how nervous systems integrate sensory information to generate behavioral outputs is key to our efforts to analyze how nervous systems function. This is a daunting challenge, given the immense complexity of most nervous systems. Even within the nervous systems of insects like *Drosophila*, there is a daunting complexity, with over 100,000 neurons and 100s of cell types that can make analysis very difficult. Despite this challenge it is possible to build an understanding of how a system works if it can be broken into simple modules that can be placed into a functional hierarchy. The power of this type of approach is evident in the vertebrate spinal cord, where the complex sensory motor circuitry that controls locomotion is generated from distinct pools of progenitor cells. These progenitors form discrete and identifiable populations of interneurons, each with defined roles in the locomotor circuits (Grillner and Jessell, 2009). Furthermore, this functional organization has a deep evolutionary significance, with progenitor domains and transcription factors that define different interneuron classes being conserved between fish and mammals (Lupo et al., 2006) despite their very different modes of locomotion.

These organizational principles are similar to the insect ventral nervous system (VNS), which, like the spinal cord, is responsible for sensory motor integration and locomotion. Work on grasshoppers and other orthopteran insects identified populations of interneurons that integrate sensory inputs into changes in posture (Burrows, 1996) and generate behaviors such as walking (Büschges et al., 2008), jumping (Heitler and Burrows, 1977a, 1977b), and flying (Robertson et al., 1982). Similar to the vertebrate spinal cord, these populations of neurons are organized along developmental principles in which specific populations arise from distinct neural precursor stem cells called neuroblasts (NBs) (Thomas et al., 1984; Truman and Bate, 1988). For example, in the grasshopper thoracic ganglia NB 4‐1 produces a population of spiking interneurons that receive primary sensory input from the legs and integrate the information to create the receptive fields of leg sensory neurons (Shepherd and Laurent, 1992; Burrows and Newland, 1993, 1994).

Within the thoracic neuromeres of the *Drosophila* VNS, most neurons are produced by a segmentally repeated and stereotyped array of 30 self‐renewing, with each of these serially homologous NBs, having a unique identity determined by position and gene expression (Skeath and Thor, 2003). Typically, each NB divides repeatedly to produce a clone of neurons that constitute its lineage. Each NB produces a chain of progenitor cells, called ganglion mother cells (GMCs). With the exception of the Type II NBs in the central brain (Boone and Doe, 2008) each GMC divides once to produce two terminally differentiated neurons or glia. The fate of the neurons is determined by Notch signaling, with one sibling activating Notch signaling and the other not; thus, each NB produces two distinct populations of neurons called hemilineages, one that is Notch “On” and the other Notch “Off” (Truman et al., 2010). Like NB4‐1 in the grasshopper, most of the hemilineages in *Drosophila* are composed of populations of interneurons that share a common anatomy and neurotransmitters (Harris et al., 2015). Truman et al. (2004) showed that despite the apparent complexity of the VNS there are essentially 33 basic projection patterns for the thousands of neurons within a thoracic hemineuromere, suggesting that, like the vertebrate spinal cord, different neuronal classes can be defined according to a developmental program. Furthermore, Harris et al. (2015) have shown that the stimulation of interneurons in a hemilineage can elicit specific and characteristic behavioral responses, suggesting that the hemilineages represent functional modules. Together, this suggests that taking a hemilineage‐based perspective on the construction of the *Drosophila* VNS will provide a deep understanding of the complex network required for processing of sensory motor information.

Despite this apparent simplicity, tracing the developmental origins of the adult hemilineages in *Drosophila* has been slow. In insects that have a complete metamorphosis, many NBs have two stages of neurogenesis. In the embryo the NBs generate a set of neurons that regulate larval behavior (Larsen et al., 2009), then after a period of quiescence the NBs reactivate for a second and longer phase of proliferation to produce the adult‐specific set of neurons. These adult‐specific neurons extend a primary neurite into the neuropil but arrest until the onset of metamorphosis, when they grow rapidly to form the adult circuitry. In most cases the neurons in a specific hemilineage extend their primary neurites into a tightly fasciculated bundle with an almost invariant trajectory in the neuropil. The limited cell mixing and migration of neurons from their parent NB means that the bundled neurites of specific hemilineages can be unambiguously identified using the cell adhesion protein, neurotactin. The assignment of neurons to a specific hemilineage was achieved by referencing the primary neurites from MARCM‐labeled NB clones to the scaffold of neurotactin‐positive bundles, hereafter referred to as tracts (Truman et al., 2004). Although this provided insight into the developmental origins of secondary neurons, its impact on understanding the developmental organization of the adult VNS was limited because the neurotactin labeling was transient and disappeared early in metamorphosis, making made it more difficult to analyze the hemilineages as they matured into adult neurons. Here we reveal that, as shown for the central brain (Lovick et al., 2013), an antibody to the membrane‐associated protein neuroglian not only reveals the hemilineage scaffold in the larval VNS but also persists through metamorphosis and allows identification of the hemilineage tracts in the adult. The data show it is possible to chart these functional modules within the adult VNS and uncover the developmental rules for its assembly.

## MATERIALS AND METHODS

### Fly stocks

All flies were reared on standard cornmeal and molasses food at 25°C. Lines used include: GAL4 lines from the Rubin GAL4 collection (Pfeiffer et al., 2008) and OK371 (Mahr and Aberle, 2006). The lines and techniques used to “immortalize” larval hemilineages are exactly as detailed in Harris et al. (2015) and were undertaken at the same time.

### Generation of MARCM clones

The MARCM technique was used, in which the FLP/FRT system induced clones that lacked GAL80, a suppressor of GAL4, to make *CD8::GFP*‐labeled clones in an unlabeled background (Lee and Luo, 2001). The GAL4 drivers used for MARCM were *elav* ^*C155*^ and OK371. Eggs were collected on apple juice plates for 2 hours, held for 24 hours (both at 25°C), and the larvae were heat shocked between 3 and 5 hours after hatching. Larvae were reared on standard food at 25°C. Nervous systems were dissected from 1‐day‐old adults.

### Preparation and examination of tissues

Tissues were dissected in PBS (phosphate‐buffered saline, pH 7.8) and fixed in 4% buffered formaldehyde for 1 hour at room temperature. Fixed tissues were washed in PBS‐TX (PBS with 1% Triton X‐100, Sigma, St. Louis, MO), incubated in 10% normal donkey serum (Jackson ImmunoResearch, West Grove, PA; Cat. no. 017‐000‐001 RRID:AB_2337254) for up to 6 hours, and then in 1:1,000 rabbit anti‐GFP (Molecular Probes (Invitrogen), Eugene, OR; Cat. no. A11122 RRID:AB_221569), 1:40 rat anti‐N‐cadherin (Developmental Studies Hybridoma Bank, Iowa City, IA; Cat. no. DN‐Ex 8 RRID:AB_528121), and 1:40 mouse anti‐neuroglian (Developmental Studies Hybridoma Bank; Cat. no. BP 104 anti‐neuroglian RRID:AB_528402) overnight at 4°C. Tissues were washed in PBS‐TX and incubated with 1:500 AlexaFluor 488‐conjugated donkey antirabbit (Jackson ImmunoResearch; Cat. no. 715‐545‐151 RRID:AB_2341099), AlexaFluor 594‐conjugated donkey antimouse (Jackson ImmunoResearch; Cat. no. 715‐585‐151 RRID:AB_2340855), and AlexaFluor 647‐conjugated donkey antirat (Jackson ImmunoResearch; Cat. no. 712‐605‐153 RRID:AB_2340694) overnight at 4°C. This is a standard antibody protocol we use for all our studies. The N‐cadherin staining reveals the fine structure of the neuropil but the information provided by this channel was not used in this study. Tissues were washed in PBS‐TX, mounted onto poly‐lysine‐coated coverslips, dehydrated through an ethanol series, cleared in xylene, and mounted in DPX mountant (Sigma‐Aldrich). Nervous systems were imaged on either a Zeiss LSM 510 or Zeiss 710 confocal microscope at either 40× or 63× with optical sections taken at either 1 μm or 2 μm intervals. LSM files were contrast‐enhanced as necessary. *z‐*projected images were created using ImageJ (http://rsbweb.nih.gov/ij/), Z sections were made with Vaa3D (Peng et al., 2010) (http://home.penglab.com/proj/vaa3d/home/index.html) and montages made in Adobe Photoshop (San Jose, CA).

### Antibody characterization

Anti‐GFP is a commercially available polyclonal antibody raised in rabbit to purified GFP (Table 1). In our studies its specificity is validated by internal controls such that the pattern of immunostaining consistently and precisely matches the patterns of fluorescence produced by Gal4‐driven GFP expression in a vast array of different GAL4 lines including negligible immunoreactivity in non‐GFP‐expressing tissues. It has also been widely used in many other systems and organisms to successfully demonstrate the localization of expressed GFP.

Anti‐neuroglian (RRID:AB_528402) is an IgG1 antibody raised in mouse against a nervous system‐specific 180 kD splice variant of *Drosophila* neuroglian (Hortsch et al., 1990) (Table 1). The 180 kD isoform can be purified to homogeneity and the derived amino acid sequence was identical to the sequence for the amino terminus of the 167 kD isoform (Bieber et al., 1989; Hortsch et al., 1990). The antibody recognizes an epitope on the cytoplasmic segment of the long form of the protein (Hortsch et al., 1990). Null mutations in the nrg gene are lethal but hypomorphic mutations have greatly reduced expression (Hall and Bieber, 1997). Neuroglian protein expression assessed using BP‐104 in nrg^3^ mutants, which are temperature‐sensitive, shows that at the restrictive temperature, labeling in neuroglian‐positive neuronal processes was eliminated (Hall and Bieber, 1997).

## RESULTS

In this article we use both the postembryonic designations of the NBs (Truman et al., 2004) and the embryonic nomenclature of Schmid et al. (1999). The nomenclature for postembryonic hemilineages devised by Truman et al. (2010) is a notation that describes the NB of origin and whether they are the Notch‐on (A) or Notch‐off (B) daughter of the GMC division. With the embryonic nomenclature, while there is agreement in the correspondence between the embryonic and postembryonic designations in most instances, in some cases they are in dispute (Birkholz et al., 2015; Lacin and Truman, in preparation) and these have been so indicated. Identification of which hemilineages survive into the adult was based on Truman et al. (2010) describing which thoracic hemilineages survive to late third instar.

### Metamorphosis of the neuroglian scaffold

**Figure 1:**
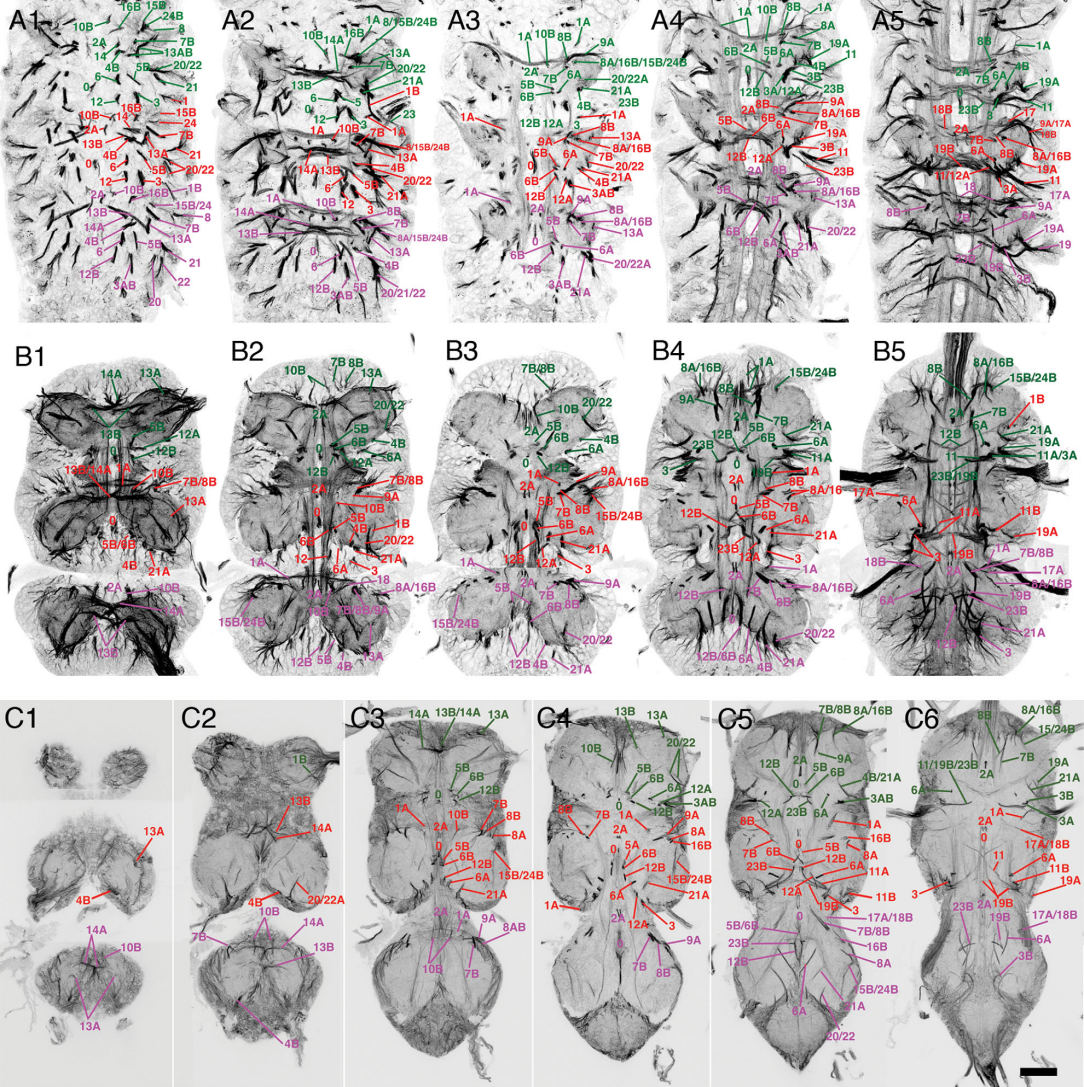
Metamorphosis of the neuroglian scaffold in the VNS. Confocal images of neuroglian‐stained hemilineage tracts at three stages of metamorphosis showing the transformation of the neuroglian scaffold from early pupa to adult. Each tract is labeled with its corresponding hemilineage notation. **(A)** Early pupa 24 hours APF. **(B)** Mid‐stage pupa 36 hours APF, **(C)** Late pupa 72 hours APF. Each panel is a composite slice of five confocal Z slices taken at different planes on the dorsal ventral axis selected to maximize the visualization of the tracts. Moving from left to right each image is taken at an increasingly more dorsal plane. Anterior is up. Scale bar = 100 μm.

Shortly after the end of larval life the postembryonic NBs in the VNS have ceased division and their progeny are clustered in clonal units in the cortical layer of the VNS. By this stage each neuron in a hemilineage has extended a primary neurite to form, along with their sibling neurons, a coherent and hemilineage‐specific neurite tract with a discrete point of entry into the neuropil and an almost invariant projection within the neuropil. Labeling the late larval VNS with anti‐neuroglian reveals the full hemilineage tract scaffold in the larval VNS and it is thereby possible to identify the tracts of each of the 33 hemilineages that contribute to each hemisegmental unit of the thoracic neuromeres (Fig. 1A1–5; Supplementary Fig. 1).

Unlike neurotactin, the neuroglian signal does not disappear at the onset of metamorphosis and continues to label the scaffold through metamorphosis and into adult stages (Fig. 1A–C). This timeline shows that most of the hemilineage tracts remain tightly fasciculated and coherent through metamorphosis. Despite the changes in the gross morphology of the VNS (expansion and rotation of the thoracic neuromeres), the hemilineage tracts retain the same relative positions and are consistent between preparations (*n* > 200). Although the majority of the neurite tracts remain labeled, a small number become weakly labeled and less distinct but still recognizable in the early adult.

By identifying each hemilineage tract at selected stages of metamorphosis, we traced all hemilineage tracts through metamorphosis into the adult for all three thoracic neuromeres. Each adult hemilineage tract can be directly related to its larval tract.

**Figure 2:**
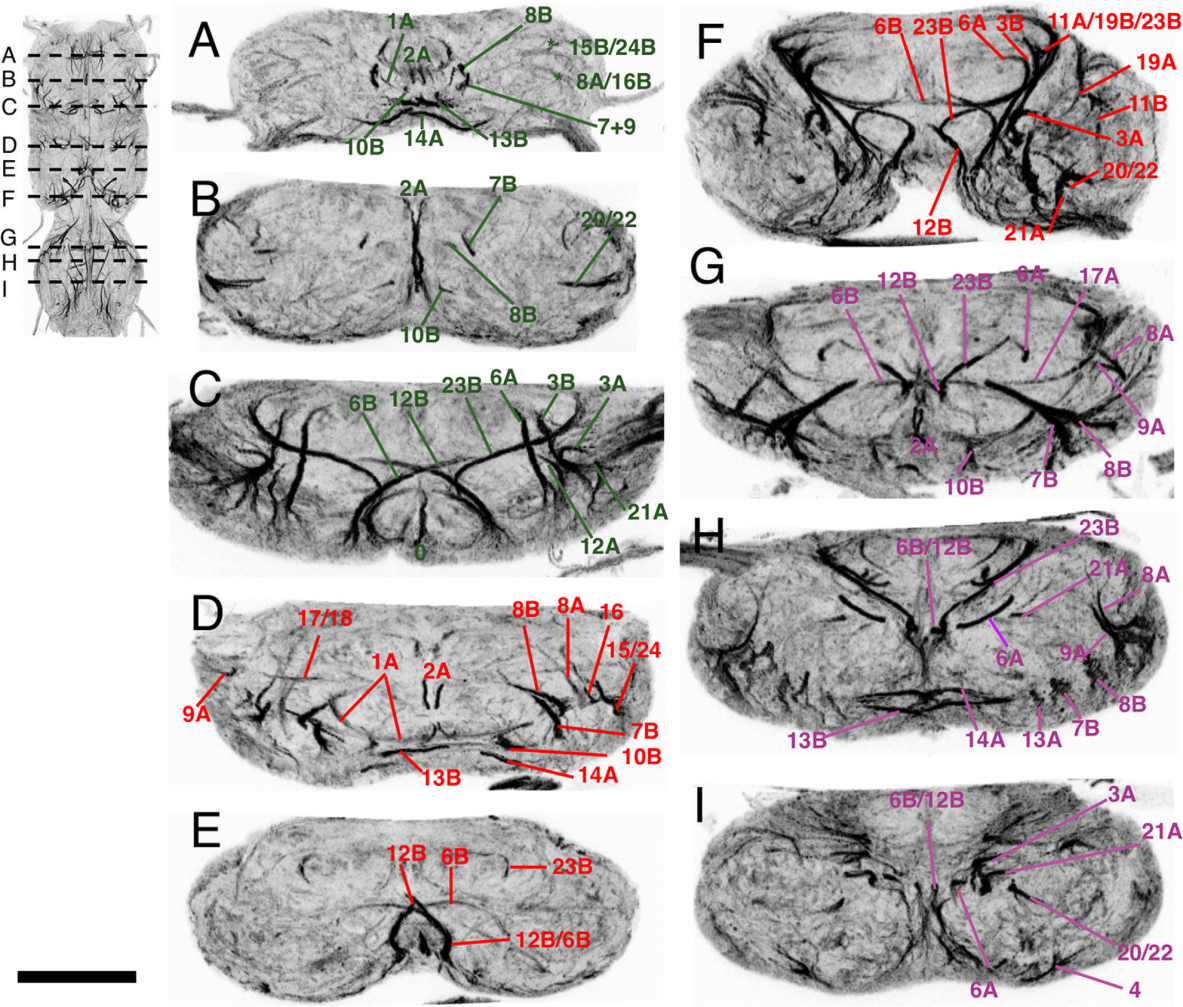
Transverse sections of the adult VNS neuroglian‐labeled hemilineage scaffold in the adult VNS. Each panel is a 5‐μm‐thick optical slice through the same confocal stack at a position to give the most complete image of the scaffold organization. The sequence of images (A–I) moves from anterior to posterior with the position of each section shown in the inset figure. **(A–C)** Prothoracic neuromere. **(D–F)** Mesothoracic neuromere. **(G–I)** Metathoracic neuromere. Dorsal is up. Scale bar = 100 μm.

In early pupa (∼24 hours after pupa formation [APF], Fig. 1A; Supplementary Fig. 2), the neuroglian‐labeled scaffold is identical to the larval neurotactin scaffold and it is possible to identify all tracts found in the larva (Truman et al., 2004). By mid‐stage (∼36 hours APF, Fig. 1B; Supplementary Fig. 3), the VNS has begun to acquire its adult shape with the expansion of the three thoracic neuromeres. There are only slight changes in the scaffold and one can still identify all larval tracts. By later stages (>70 hours APF, Fig. 1C; Supplementary Fig. 4) the VNS has the distinctive adult shape; although some tracts are distorted by morphological changes, they retain their relative positions and can be readily identified. For example, the tracts from the medial hemilineages 0, 2A, 5B, 7B, 10B, 14A, 15B, 16B, and 23B are almost unchanged throughout (Figs. 1A–C, 2). A small number of hemilineages, however, showed significant changes.

#### Anterior prothoracic lineages

At the anterior of the prothoracic neuromere the distortion caused by the anterior migration of the gnathal neuromeres and the formation of the cervical connective results in the anterior hemilineages (7B, 8B, and 9A) being drawn anteriorly, making the identification of the tracts more difficult. In early pupa the 7B, 8B, and 9A tracts are easily distinguishable (Fig. 1A), but in the adult the tracts fuse to form a single tract (Fig. 1B). The 7B and 8B tracts are only distinguishable at the point when 8B defasciculates and turns medially just anterior to the 2A tract and 7B projects posteriorly (Fig. 1C5–6). The 9A tract enters the neuromere from a dorsal position to fuse with 7B and 8B with 9B only distinguishable by its dorsal origin (Fig. 1B4).

#### Lateral lineages

The lateral hemilineages are displaced due to expansion of the neuropil and lie on the dorsal surface of the adult VNS. Despite the displacement they retain their relative positions with the anterior lateral lineages 9A, 17, and 18 and the posterior lateral hemilineages 11, 19, and 23B retaining their close associations to form two distinct clusters. The tracts from the hemilineages in each cluster, however, are fused into a single tract and individual projections can only be resolved as they defasciculate, e.g., hemilineages 11, 19, and 23 (Fig. 1C6) and 17B and 18B (Fig. 1C5–6).

#### Separation of sibling hemilineages

With lineages in which both the A and B hemilineages survive, the two hemilineages separate to form two distinct projections. At the start of metamorphosis sibling hemilineages are typically closely associated as a single cluster with neurites sharing the same or adjacent entry points into the neuropil. As the VNS expands the two hemilineages pull apart and can lose obvious anatomical association. This is most extreme for the hemilineages of lineages 1, 8, 12, and 13. In all cases such changes are consistent between preparations.

### Validation of the hemilineage identification

Although we traced the adult neuroglian tracts through metamorphosis, we also independently verified the identity of each. Tracing GAL4 lines with expression in known larval hemilineages through metamorphosis proved unsuccessful. A screen of expression in over 7,000 GAL4 lines (Li et al., 2014) resulted in a collection of lines with expression in single larval hemilineages, but analysis of the adult expression showed that in almost every case the expression changed during metamorphosis, with adult expression showing no relationship to that in the larva. We identified only two lines with a pattern of GAL4 expression that was hemilineage‐specific and retained in adult stages. These lines are OK371 and R52B04, both of which were used in this study.

In the absence of GAL4 lines with expression in single hemilineages in larva and adult, we made use of techniques developed to maintain and “immortalize” larval hemilineage‐specific expression to preserve the larval expression pattern into the adult (Harris et al., 2015). This provided a library of crosses that gave a permanent hemilineage‐specific marker that can be detected in the adult and allow confirmation of the hemilineage identity independent of the neuroglian tract time series.

### Anatomy of the adult neuroglian scaffold

Below is a description of the adult neuroglian scaffold to provide an anatomical framework that allows identification of each hemilineage. Rather than discuss lineages in number order, which has no functional basis, we present the lineages based on their anatomy.

#### Lineages that contribute to the anterior commissure

In the larva, axon bundles from seven lineages run through the anterior commissure (Truman et al., 2004). Three (1A, 13B, and 14) contribute to the ventral anterior (vA) commissure, while four (7, 8B, 10, and 18) run in the intermediate anterior (iA) commissure. Along the anterior–posterior axis the vA and iA commissures are separated by the bundles from lineage 2. Ventrally, the lineage 1 bundle is anterior to lineage 2 tracts and the 13 and 14 bundles are posterior, with 14 anterior to 13. At the intermediate level, the tracts from lineages 10 and 18 are anterior to lineage 2, while 7 and 8 are posterior to lineage 2 with 7 posterior to 8. The relative typography persists through metamorphosis and in the arrangement of the adult tracts.

##### Lineage 1 (NB 1‐2)

**Figure 3:**
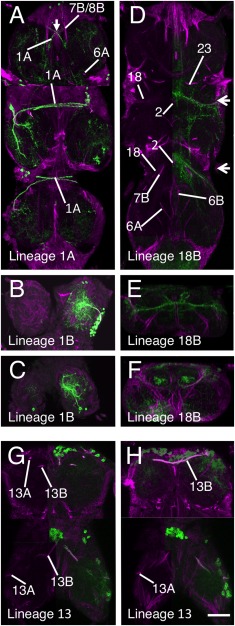
Validation of the hemilineage tract identification for the lineages that contribute to the anterior commissures. Each panel is a composite of five horizontal Z stacks to best illustrate the identification. Where possible, the left side of the image has the GFP signal (green) removed to allow identification of the underlying neuroglian signal (magenta). **(A–C)** Lineage 1 revealed by immortalization of the larval expression pattern of line R22G11. (A) Hemilineage 1A. (B,C) Hemilineage 1B tract in T1 (B) and T2 (C). **(D–F)** Hemilineage 18B revealed by immortalization of the larval expression pattern of line R27A09. (D) Hemilineage 18B projections in T2 and T3. (E,F) Transverse sections E (T2) and F (T3) at the levels indicated by the arrows in D. **(G,H)** Lineage 13 revealed by immortalization of the larval expression pattern of line R81F02 in T1 and T2 only. Scale bar = 100 μm.

Lineage 1 is the anteriormost lineage in each neuromere and is the only lineage that projects fibers into two neuromeres (Truman et al., 2004). In the larva, the 1A neurons run in the vA commissure of the neuromere of origin, while the 1B neurons project into the next anterior segment. In the larva and early pupa, the cells of the 1A and 1B hemilineages are closely associated (Figs. 1A1, 2) and their tracts share a common point of origin (Figs. 1A1, 2). As the VNS expands the 1A and 1B cells separate, the 1A cells move dorsally to the dorsal third of the VNS, and the 1B cells are drawn anteriorly to sit at the posterior margin of the next anterior segment (Fig. 3B–C) into which they project their primary neurites. Thus, the projections of the sibling lineage 1 hemilineages and their cells are in different neuromeres.

In adult T1, the 1A tract is weakly labeled and barely detectable, although it can be seen at earlier stages (Fig. 1). The R22G11 GAL4 line allows identification of the 1A tract in T1 (Fig. 3A). This shows 1A enter the neuropil from an anterior position adjacent to the midline to form a tract that passes ventrally under the axons of the cervical connective to cross the midline. In T1, 1A forms a cruciform structure with its contralateral homolog (arrow in Fig. 3A). In T2, the 1A tract is strongly labeled at all stages and projects ventromedially (Fig. 1C3–4), to cross the midline as part of a superficial anterior ventrally located commissure (Figs. 1C3, 3A). In T3, 1A is less obvious than in T2 and projects anteromedially to cross the midline in a superficial anterior–ventral commissure (Figs. 1C3, 3A).

The neurons of the 1B hemilineage die in T1, but persist in T2, T3, and A1 to innervate the leg neuropil of the segment anterior to their origin. The 1B neurons in T2 form a short neuroglian‐positive tract (Fig. 3B) that runs superficially around the rim of the neuropil to enter the T1 neuropil ventrally in the posterior lateral quarter of the neuromere and projects medially (Fig. 3B). The 1B neurons from T3 project into the T2 leg neuropil and are not strongly labeled and cannot be readily identified from the neuroglian signal (Fig. 1C). Based on GFP expression (Fig. 3C), the T3 cells enter the T2 neuropil ventrally and project medially.

##### Lineage 7 (NB 4‐2 or 3‐2)

**Figure 4:**
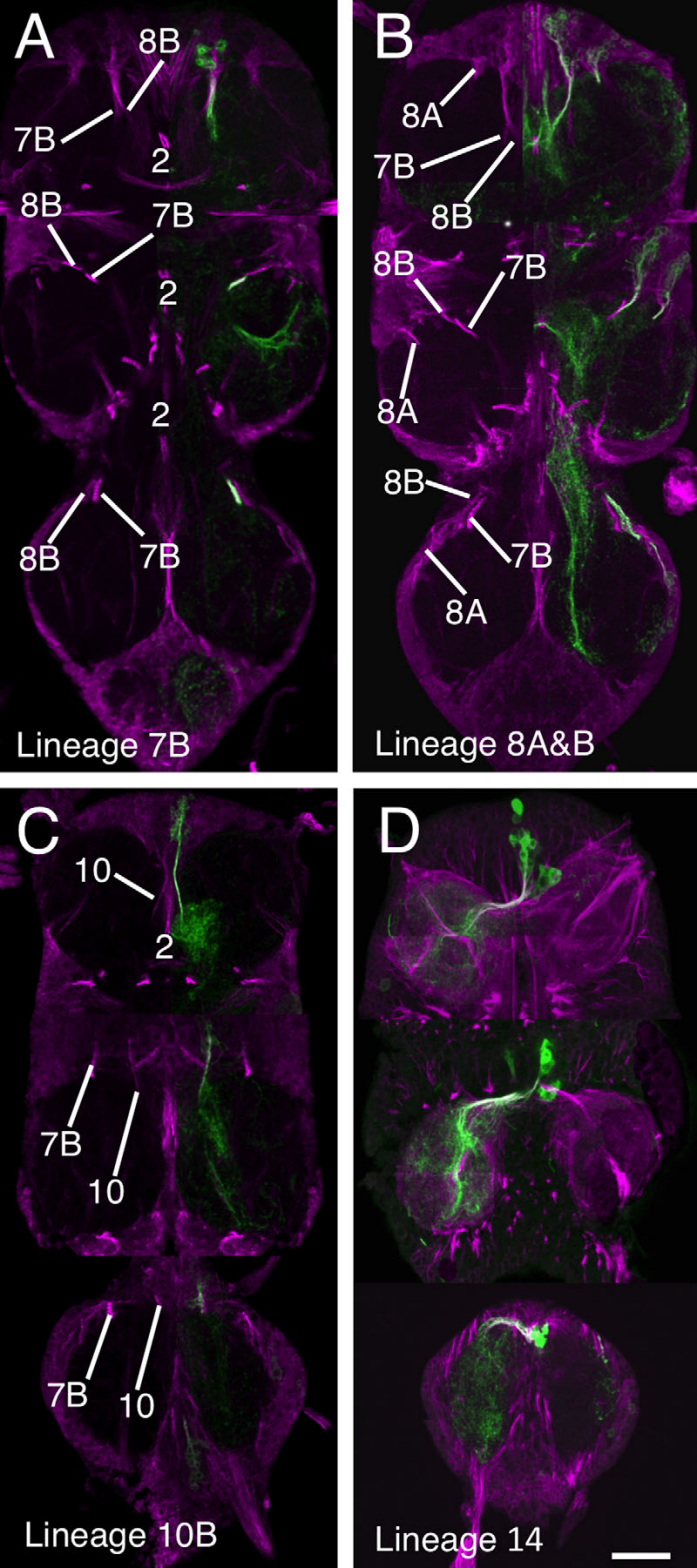
Validation of the hemilineage tract identification for lineages that contribute to the anterior Commissure. Data organization as in Fig. 3. **(A)** Hemilineage 7B revealed by immortalization of the larval expression pattern of line R75H06. **(B)** Lineage 8 revealed by immortalization of the larval expression pattern of line R30C03. **(C)** Hemilineage 10B revealed by immortalization of the larval expression pattern of line R13B08. **(D)** Hemilineage 14B revealed by the expression pattern of line OK371. Scale bar = 100 μm.

Only hemilineage 7B is found in the adult (Truman et al., 2010) and is easily identifiable in T2 and T3 throughout metamorphosis (Fig. 1A–C). In T1 the 7B neurons are displaced anteriorly and the 7B tract enters the neuropil along with the 8B and 9 axons as a single, fused, posteriorly directed tract (Fig. 4A). The 7B axons can only be recognized as they diverge when 8B turns medially and 7B continues posteriorly (Figs. 1C6, 4A). In T2 and T3 the 7B projections are easily distinguished (Fig. 4A), with 7B running parallel and posterior to 8B (Figs. 1, 4A), a feature that persists from the larva. Identification of the adult tract was confirmed by immortalizing the expression of the R75H06 line (Fig. 4A).

##### Lineage 8 (NB 2‐4 or 3‐3)

Hemilineages 8A and 8B are both found in the adult and their identification was confirmed by the immortalized expression of GFP with the R30C03 cross (Fig. 4B). The tracts from each hemilineage are visible in all three thoracic neuromeres and throughout metamorphosis (Fig. 1A–C). In early pupa both tracts share a common entry point into the neuropil and pass through the lateral cylinder (Fig. 1A3). As they pass through the cylinder 8A turns laterally and 8B turns medially (Fig. 1A4). By mid‐pupa the tracts have separate entry points into the neuropil (Fig. 1B) with 8B medial to 8A and with 8B projecting medially and 8A projecting laterally (Fig. 1B3–5).

In adult the 8A tract projects to the ipsilateral leg neuropil and is closely associated with the 16B tract, which enters the neuropil just lateral to the 8A tract (Fig. 4B). In adult T1 we cannot use neuroglian staining to resolve 8A from 16B as they form a single tract that projects posteriorly into the ipsilateral neuropil. In adult T2 and T3, 8A and 16B enter the neuropil at the same point as a single tract and project medially before they diverge with 8A, taking the more posterior pathway (compare Figs. 4B and 6H).

The adult 8B hemilineage is fasciculated with the 7B neurons in anterior T1 at their point of entry to the neuropil, but 8B defasciculates and turns medially just anterior to the 2A projection (Fig. 4B). In T2 and T3 the 8B tract, along with 7B, forms a characteristic medially projecting doublet of projections with 8B the anteriormost (Figs. 1C5, 4B).

##### Lineage 10 (NB 2‐2)

Hemilineage 10A neurons die in the larva, while the 10B neurons project through the iA commissure anterior to the 2B bundles (Truman et al., 2010). The 10B tract can be identified in all thoracic neuromeres and all stages of metamorphosis (Fig. 1A–C). Immortalized GFP expression with line R13B08 provides a strong and clean signal, confirming the identity of the neuroglian tract (Fig. 4C). In T1 the 10B cells are drawn anteriorly and the tract enters the neuropil close to the cervical connective to form a bowed projection that runs posteriorly and lateral to 2A (Figs. 1C4, 4C). The 10B insertion can be distinguished from 8B, 7B, and 9 by its more medial and ventral position (Fig. 2A). In T2 and T3 the 10A bundle is short and only the ventral most parts are labeled as it projects dorsally into the neuropil just lateral to 2A (Figs. 1C3, 2D,F, 4C).

##### Lineage 13 (NB 3‐3 or 4‐2)

In the larva, the 13A siblings project to the ipsilateral leg neuropil while the 13B siblings project across the vA commissure to the contralateral leg neuropil. Immortalized expression of GFP with R81F02 confirmed the identity of both 13A and 13B tracts (Fig. 3G,H) in the adult. As with lineage 1, the two hemilineages separate during metamorphosis. At 12 hours APF the neuroglian tracts from each hemilineage diverge from a common origin in all three thoracic neuromeres (Fig. 1A1). By 36 hours APF the two hemilineage tracts have separated; the 13B cells now sit at the ventral midline and project neurites across the midline and the 13A cells have moved laterally and project neurites in an anterior lateral direction (Fig. 1B1). By 72 hours APF the hemilineages are completely separated and show no obvious anatomical associations (Fig. 1C). On occasion, the somata of the 13B neurons are drawn across the midline and can be ipsilateral to their primary projections. The 13B tract is the posterior of the two ventrally located neuroglian labeled tracts that cross the midline in the anterior of each neuromere (Figs. 1C, 2A,D,H, 3G,H).

The adult projections of the 13A neurons are similar in all three thoracic neuromeres. The neuroglian tract is short and only detectable around the entry into the neuropil (Figs. 1C1–3, 3G). It is one of the ventralmost tracts and projects dorsally. In T1, 13A enters the neuropil from a ventrolateral position at the anterior of the neuromere just lateral to the T1 leg nerve (Figs. 1C3, 3G). In T2, 13A is the ventralmost neuroglian landmark and projects along the edges of the neuropil (Figs. 1C1, 3G,H). In T3, the 13A neurons project dorsally from the ventral surface from a position in the middle of the neuromere (Figs. 1C1, 2H).

##### Lineage 14 (NB 4‐1)

In larva, almost all the 14B neurons die, while the 14A neurons persist and project across the vA commissure to the contralateral neuropil (Truman et al., 2010). The 14A tract can be recognized at all stages of metamorphosis (Fig. 1A–C) and its position remains relatively unchanged throughout. 14A is the anterior of two neuroglian‐positive bundles that form the vA commissure (Fig. 1C2–3, 2A,D,H). The other is from 13B.

Lineage 14 is revealed by line OK371, which has lineage 14‐specific expression from larva to adult and allows lineage 14 to be traced through development without the need for immortalization. The expression confirms the identification of the 14A tract (Fig. 4D). The very few surviving postembryonic 14B neurons project dorsally through the lateral cylinder and cross the midline close to the dorsal surface. They do not have a tract that can be detected by neuroglian labeling.

##### Lineage 18 (NB 3‐4 or 2‐4)

Only hemilineage 18B is found in the adult (Truman et al., 2010). The hemilineage is absent from T1 and in T2 and T3 its cells lie at the dorsal, anterior edge of the neuromere. The 18B tract can be identified throughout metamorphosis (Fig. 1A–C). In early and mid‐pupa 18B enters the neuropil from a lateral position and is difficult to discriminate from the closely associated 9A and 17A tracts (Figs. 1A4–5, B4–5). In adult, 18B enters the neuropil from a dorsal lateral position and projects medially at a mid dorsoventral plane (Fig. 1C5–6). Immortalization of 18B expression in line R27A09 confirmed the adult hemilineage 18B trajectory (Fig. 3D–F), showing that it projects medially to cross the midline anterior to the 2A tracts, consistent with its larval projection (Fig. 3D).

#### Lineages that contribute to the posterior commissure

Neurite bundles from five lineages contribute to the posterior commissure in the larva. Three (5, 6, and 12) approach the intermediate posterior (iP) commissure from a ventral location, forming a structure termed the “ventral arch,” and two (19 and 23) approach from lateral or dorsal locations. Lineage 6 also contributes a hemilineage (6A) that joins the dorsal posterior (dP) commissure (Truman et al., 2004). The larval topology of the posterior commissure persists into the adult.

##### Lineage 5 (NB 5‐3)

**Figure 5:**
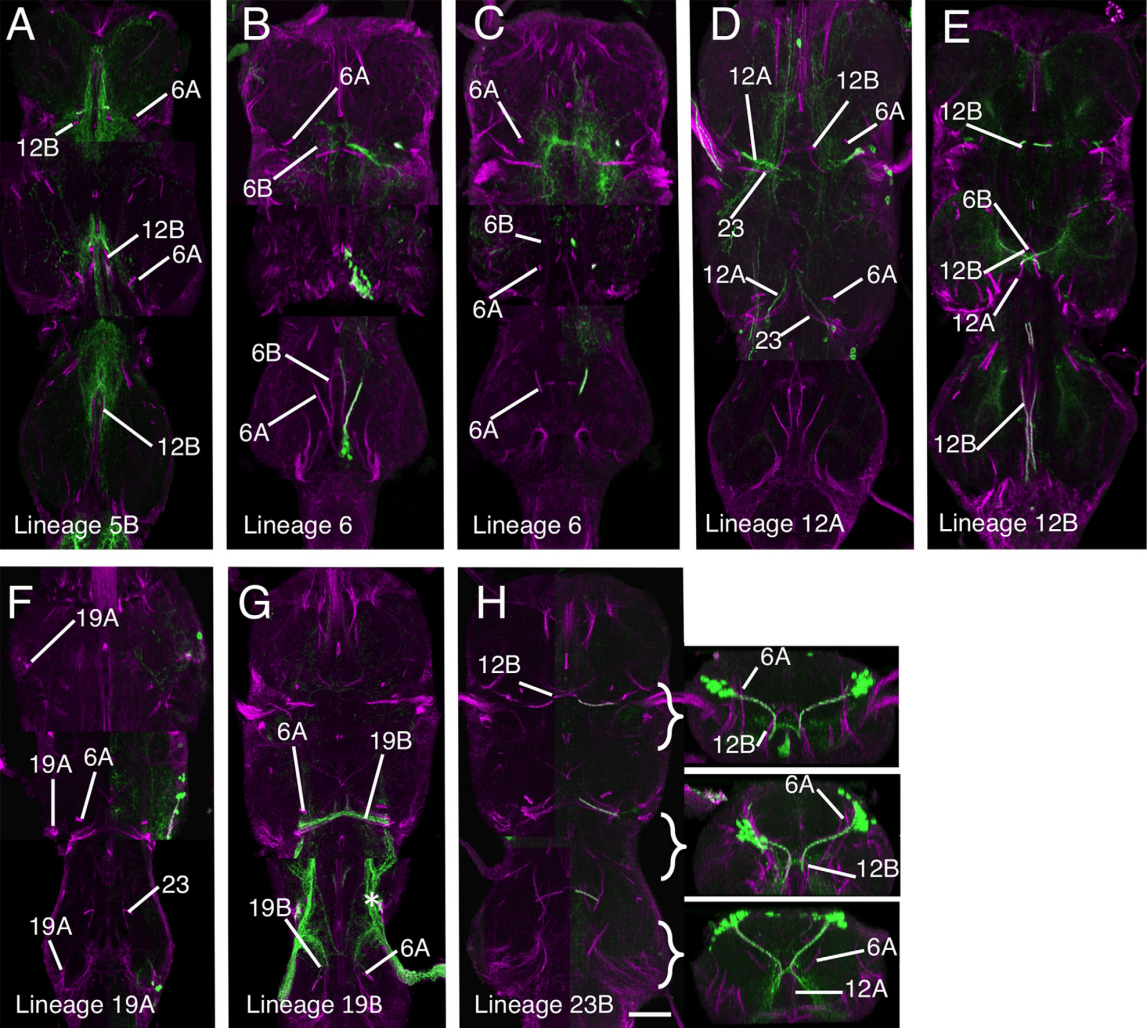
Validation of the hemilineage tract identification for lineages that contribute to the posterior commissure. Data organization as in Fig. 3. **(A)** Hemilineage 5B revealed by immortalization of the larval expression pattern of line R86D02. **(B,C)** Lineage 6: (B,C) are at different planes on the dorsal ventral axis to illustrate the differing trajectories of the 6A and 6B hemilineages with C being dorsal to B. **(D,E)** Lineage 12, with the A hemilineage revealed by immortalization of the larval expression pattern of line R24B02 (D) and the B hemilineage by immortalization of the larval expression pattern of line R15D11 (E). **(F,G)** Lineage 19 with the A hemilineage revealed by immortalization of the larval expression pattern of line R84E06 (F) and the B hemilineage by immortalization of the larval expression pattern of line R50C03 (G) (* indicates the projections of sensory afferents from the halteres). **(H)** Hemilineage 23B revealed by immortalization of the larval expression pattern of line R77C10 with transverse sections at the levels indicated. Scale bar = 100 μm.

In the larva, the 5A neurons die and the 5B neurons form the anteriormost bundle of the ventral arch (Truman et al., 2010). In early metamorphic stages the 5B tract is strongly labeled, compact, and recognizable (Fig. 1A1–2,B1–2) in all thoracic neuromeres. In adult, 5B has a less compact structure and is still the anteriormost of a trio of medial projections along with 6B and 12B that converge on the midline (Fig. 1C3–5). Immortalizing the larval 5B expression using line R86D02 provides a strong and clean signal that confirms its identity (Fig. 5A).

##### Lineage 6 (NB 5‐2)

In the thoracic neuromeres both 6A and 6B hemilineages survive in the adult. The tracts from both hemilineages project dorsally in all three thoracic neuromeres, with 6B crossing the midline as the middle of the trio of tracts of the ventral arch (Figs. 1C3, 4). 6B is visible at all stages of metamorphosis, with a similar pattern in all three thoracic neuromeres (Fig. 1A–C). As in larva, 6B is posterior to 5B throughout its length, and extends more dorsally. In T3, 5B and 6B are closely associated and it is difficult to resolve the separate tracts for most of their length (Figs. 1C5, 5B).

Hemilineage 6A produces one of the dorsalmost projecting tracts in the adult (Figs. 1C4–5, 2, 5B–C) and forms the dorsal component of the posterior commissure (dP) (Figs. 2, 5B–C). In T1 it projects almost perpendicularly from ventral to dorsal (Figs. 1C, 2C, 5C). In T2, 6A projects laterally and dorsally (Figs. 1C, 2F, 5C). In T3, 6A projects anteriorly and dorsally and terminates in dorsal neuropil at the interface between the T2 and T3 neuromeres (Figs. 1C, 2E, 5C).

##### Lineage 12 (NB 6‐1)

Both 12A and 12B hemilineages survive in the adult in T1 and T2, but in T3 only 12B persists. The identity of 12B was confirmed by immortalized expression using line R15D11 (Fig. 5E) and 12A tract by immortalized expression with line R24B02 (Fig. 5D). In early and mid‐pupa, hemilineage 12A and 12B share a common entry point into the neuropil (Fig. 1A1–3). In adult, the 12A and 12B tracts separate into distinct tracts with no physical association with 12B projecting ventrally and medially and 12B projecting dorsally and laterally (Fig. 1C4).

In all thoracic neuromeres 12B forms the posteriormost part of the ventral arch, along with 5B and 6B (Fig. 1C4–5). In T3, 12B follows the same projection but for most of its proximal segment it is not possible to distinguish it from 5B and 6B. The three can only be distinguished at the distal points as they separate prior to crossing the midline.

In T1 and T2, 12A projects into dorsal neuropil and is readily detectable in both neuromeres (Figs. 1C4–5, 2C–F, 5D). In T1, 12A projects medially and dorsally (Fig. 1C) between 6A and 3 and passing ventrally under 23B (Fig. 2C) to merge with the tract from 11A. In T2, 12A also projects dorsally and laterally, but is more anteriorly inclined than its T1 counterpart (Figs. 1C4–5, 5D).

##### Lineage 19 (NB 6‐2)

Both 19A and 19B hemilineages are found in the adult but from the larval stages the two hemilineages form separate and distinct projections into the neuropil. Both hemilineages are evident in all three thoracic neuromeres at all stages of metamorphosis. Immortalized expression of GFP from larval‐specific hemilineages using lines R84E06 (for 19B) and R50C03 (for 19A) was used to confirm the 19A and B identities (Fig. 5F–G). Expression in line R84E06 also includes strong expression in haltere sensory afferents (asterisk in Fig. 5G) but the 19B projections can be identified.

In adult T1, the 19A tract enters the neuropil in the dorsal posterior quarter completely separate from 19B and projects ventrally and medially into posterior ipsilateral neuropil (Fig. 1C6). In T2, the neuroglian‐labeled tract is short and only evident around the point of entry into the neuropil in the posterior dorsal quarter (Figs. 1C4, 5F). In T3, the 19A entry point into T3 neuropil is at the posteriormost point of the T3 neuropil entering from the dorsal surface and projects anteriorly and ventrally (Fig. 5F).

In T1, hemilineage 19B contains only a few cells, and its tract is not detectable as a single tract as it enters the neuropil as small part of a fused tract with 11A and 23B (Fig. 1C6). In T2, the 19B tract is easily detected as a strongly labeled tract that enters the neuropil dorsally at the posterior margin of the neuromere and projects medially to cross the midline at the posterior margin of the T2 neuropil (Figs. 1C6, 5G). In T3, the 19B tract is not as strongly labeled as in T2 but it is readily detectable as it enters the dorsal neuropil and projects medially and anteriorly alongside 23B (Figs. 1C6, 5G), but there are many fewer neurons in this tract compared to T2.

##### Lineage 23 (NB 7‐4)

In the adult, only hemilineage 23B survives (Truman et al., 2010). It is identifiable at all stages of development and is one of the most characteristic features of the adult scaffold (Fig. 1A–C). In adult T1, 23B enters the VNS dorsally as part of shared tract with 11 and 19 (Fig. 1C6). As it crosses the midline 23B turns ventrally to merge with 12B (Figs. 1C5–6, 2B, 5H). In T2, 23B enters the VNS dorsally and projects medially but unlike in T1, 23B is distinguishable from 11B and 19B. As 23B nears the midline it turns anteriorly and ventrally to meet the dorsally projecting 12B to form a fused 12B/23B bundle that crosses the midline (Figs. 1C5, 2F, 5H). In T3, 23B enters from dorsal and projects medially and ventrally to meet the dorsally projecting 12B (Figs. 1C5–6, 2H, 5H). 23B is one of the only bundles to transit the neuropil dorsal to ventral, rather than ventral to dorsal (see transverse sections in Fig. 5H). It is the single longest dorsoventral tract in the neuropil and forms an important landmark in the medial cluster formed by 12B, 6B, and 5. Immortalization of GFP expression in hemilineage 23B with line R77C10 confirms its identity (Fig. 5H).

#### Leg neuropil hemilineages

Eighteen hemilineages contribute neurons to each hemisegmental leg neuropil. Half of these (1A, 1B, 8A, 12B, 13A, 13B, 19A, 23) are either commissural neurons or siblings of commissural cells and are dealt with above. The remaining leg hemilineages are detailed below.

##### Lineage 3 (NB 7‐1)

Both 3A and 3B hemilineages are found in the adult and the tracts of both hemilineages are evident throughout metamorphosis. Validation of the tracts for lineage 3 was achieved by immortalization of the larval expression of line R31H10 (3A) and R41G09 (3B). The cells of both hemilineages are displaced in the adult and these lines enabled us to identify the complex projections of both hemilineages. The 3A and 3B tracts enter the neuropil at the same point (Figs. 1C4–6 and 6A–D) and project together dorsally to the mid‐neuropil, where they diverge (Figs. 1A3–4,1B3–4, 2C,F,I, 6A–D). The 3B neurons innervate the leg neuropil and their axons bend laterally to the dorsal ipsilateral leg neuropil. They are similar in all thoracic leg neuropils.

**Figure 6:**
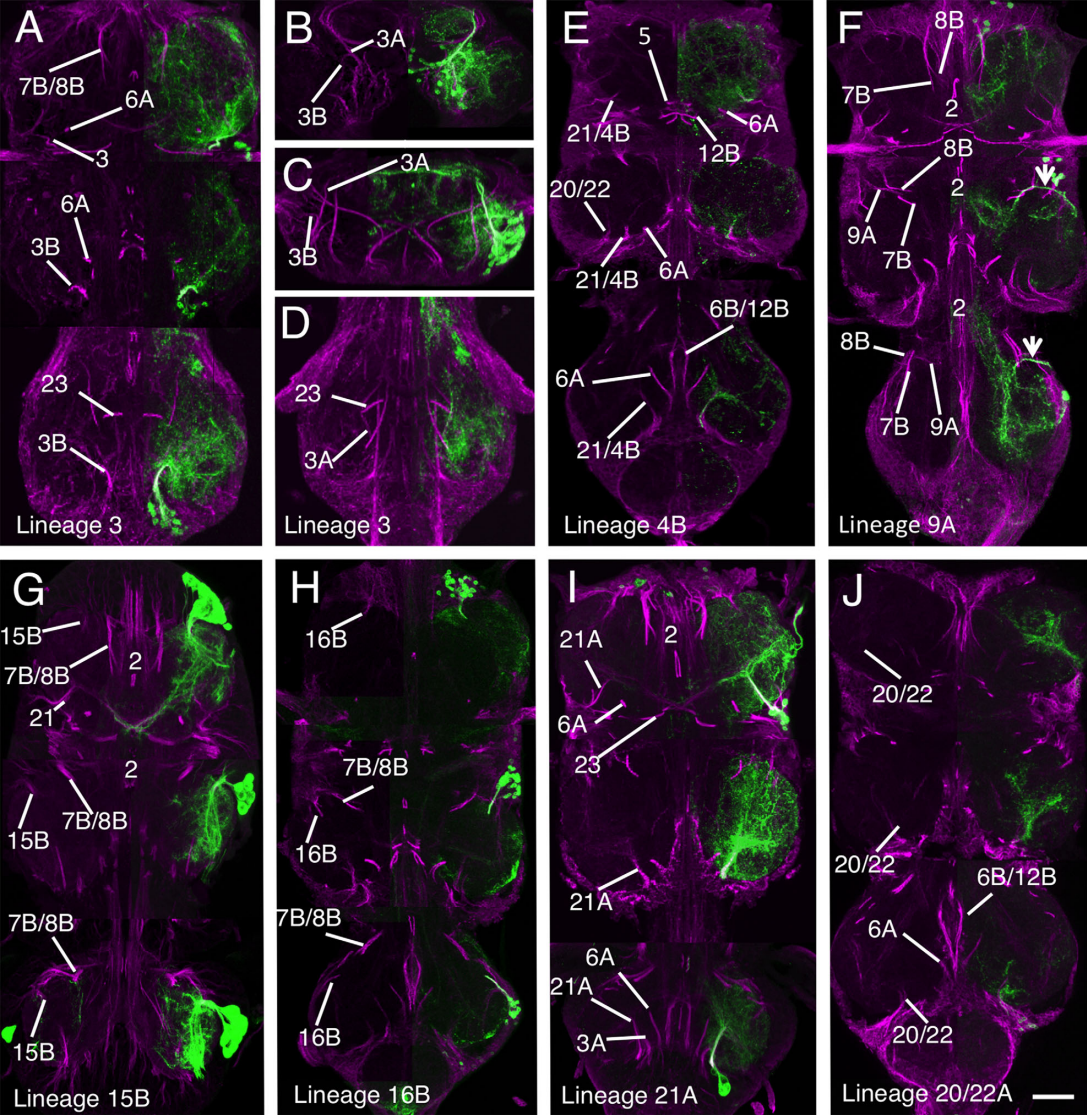
Validation of the hemilineage tract identification for hemilineages associated with the leg neuropil. Data organization as in Fig. 3. **(A–D)** Lineage 3 revealed by an *elav*‐MARCM clone of lineage 3 containing both the A and B hemilineages. (A) Horizontal section showing lineage 3 projections in T1–T3. (B,C) 5 μm transverse optical transverse sections through the stack in panel C in T1 (B) and T2 (C) to illustrate the different tracts associated with lineage 3. (D) Horizontal plane of lineage 3 in T3 from a more dorsal plane than in A. **(E)** Hemilineage 4 revealed by immortalization of the larval expression pattern of line R51D02. **(F)** Hemilineage 9A revealed by immortalization of the larval expression pattern of line R48H02. **(G)** Hemilineage 15B revealed by the expression pattern of line OK371, **(H)** Hemilineage 16B revealed by the expression pattern of line OK371. **(I)** Hemilineage 21A revealed by the expression pattern of line OK371. **(J)** Hemilineage 20/22A revealed by immortalization of the larval expression pattern of line R19H10. Scale bar = 100 μm.

The axons of the 3A neurons continue dorsally after diverging from the 3B cells and project into the dorsal flight neuropil. The 3A tracts in T1 and T2 are large and strongly labeled and terminate in dorsal neuropil (Fig. 6B,C). In T3, the 3A tract contains a much‐reduced number of fibers, produced by neuronal death during metamorphosis, and project anteriorly into dorsal T2 (asterisk in Fig. 6D).

##### Lineage 4 (NB 3‐1)

Only hemilineage 4B is present in the adult (Truman et al., 2010). In early pupal stages the 4B projection is easily recognized as a strongly labeled, medially originating, and dorsally projecting tract (Fig. 1A3–4). In later pupal and adult stages the 4B the tract is very difficult to detect from the neuroglian signal alone. Identification of 4B in later stages was heavily dependent on the immortalized expression of line R51D02 (Harris et al., 2015), which provides a weak but detectable signal that confirms the posterior displacement of the 4B cells and its close association with the hemilineage 21 tract with the 4B axons projecting anteriorly into the lateral region of ipsilateral leg neuropil (Fig. 6E).

##### Lineage 9 (NB 3‐5)

Only hemilineage 9A is evident in the adult (Truman et al., 2010). The 9A tract is evident in all three thoracic neuromeres at all stages of metamorphosis (Fig. 1A–C). In the adult, 9A is easily identified and confirmed by immortalization with line R48H02 (Fig. 6F). In T1, the 9A tract enters the neuropil from an anterior position alongside 7A and 8B. It is distinguishable as the fine branch that defasciculates and turns laterally at the point at which 8B turns medially, anterior to the 2A tract (Fig. 1C5). In T2 and T3, 9A resembles the larval projection and projects medially and ventrally to the midline with the characteristic arc anterior to 7A and 8B (Fig. 1C3–4 and arrows in 6F).

##### Motor lineages: lineages 15 (NB2‐3) and 24 (NB4‐4)

The lineage 15 NB produces both motoneurons and glia, with the 15A siblings being glia (Baek et al., 2013), and the 15B daughters becoming motoneurons (Truman et al., 2010). Hemilineage 15B is revealed by line OK371, which has lineage‐specific expression that can be readily traced through metamorphosis (Fig. 1A–C). In early (Fig. 1A2–4) and mid pupa (Fig. 1B2–4) the 15B tract is strongly labeled for neuroglian but in adult the 15B tract is only weakly labeled, but its entry into the neuropil can still be seen in all thoracic neuromeres simply due to its large size and structure (Figs. 1C3–6 and 6G). The 15B tract merges with the 24B tract to form a single tract which projects in a posterior medial direction from the point of entry to the neuropil (Fig. 6G).

Lineage 24 was not uncovered by Truman et al. (2004) and was described by Brown and Truman (2009). In the adult, only hemilineage 24B survives (Truman et al., 2010) and produces leg motor neurons that innervate proximal leg segments (Baek and Mann, 2009; Brierley et al., 2012). The adult morphology of 24B is in accord with other descriptions of leg motor neurons (e.g., Nässel et al., 1986), and is similar to, but distinct from, hemilineage 15B in both larva and adult, The adult lineage 24 neuroglian tract is closely associated with its 15B counterpart and is indistinguishable from it.

##### Lineage 16 (NB 1‐1)

Only hemilineage 16B is found in the adult and is revealed in OK371, and can be directly traced through metamorphosis (Fig. 6H). The 16B tract can be readily identified in early (Fig. 1A3) and mid‐pupa (Fig. 1B4 and 5). In the adult it is closely associated with 8A (compare Figs. 4B and 6H). In T1, it is not possible to distinguish the 16B and 8A tracts (Figs. 1C5–6, 4B, 6H). In T2 and T3, though, 8A and 16B enter the neuropil as a single tract and project medially but then diverge with 16B taking a more anterior pathway (Figs. 1C5 and 6H).

##### Lineage 21 (NB 4‐3)

Hemilineage 21A is a moderate‐sized cluster of local interneurons, while 21B consists of a couple of motoneurons, with the remainder of that lineage undergoing programmed cell death (Truman et al., 2010). The 21A tract is identifiable at all stages of metamorphosis (Fig. 1A–C) and is revealed in line OK371, making it possible to trace the lineages directly from larva to adult (Fig. 6I). 21A has the same morphology in all three thoracic neuromeres and arises in the posterior quarter just medial to 20A/22A and projects anteromedially into ipsilateral neuropil (Fig. 6I). In all thoracic neuromeres the tract shows a characteristic “T”‐shaped bifurcation producing separate medial and lateral projections (Figs. 1A–C and 6I).

##### Lineages 20/22 (NB 5‐4; 22 is a new: NB 5‐4b)

In the larva, 20 and 22 are neighboring lineages whose progeny show almost identical projection patterns into the leg neuropil. Both consist almost entirely of the A hemilineages, with the B hemilineage represented by one or two motoneurons with the remainder dying (Truman et al., 2010; Brierley et al., 2012). We have been unable to distinguish the two hemilineages in the adult and consequently treat them as a single hemilineage. At all stages of metamorphosis the 20A/22A cells remain closely associated in the posterior lateral quarter of all thoracic neuromeres (not shown). The identification of 20A/22A was confirmed by immortalized expression of R19H10 line, which expresses in the larval 20A/22A hemilineages (Fig. 6J).

The 20A/22A tract is readily identified at all stages of development (Fig. 1A–C). In the adult, the entry of the 20A/22A tract rotates with the segmental rotation of the leg neuropil. In T1 the tract is lateral and ventral to 21A (Figs. 1C4–5, 6J), entering the neuropil just posterior to the T1 leg nerve, projecting anteriomedially to terminate in ipsilateral neuropil (Fig. 8C). In T2, 20A/22A enters the neuropil lateral to 4A and ventral to 21A and projects anteriorly to terminate in ipsilateral neuropil (Figs 1C2–3, 6J). In T3, 20A/22A is the posteriormost tract, entering posterior neuropil from a medial position (Figs. 1C5 and 6J).

#### Miscellaneous lineages

##### Lineage 2 (NB 2‐1)

**Figure 7:**
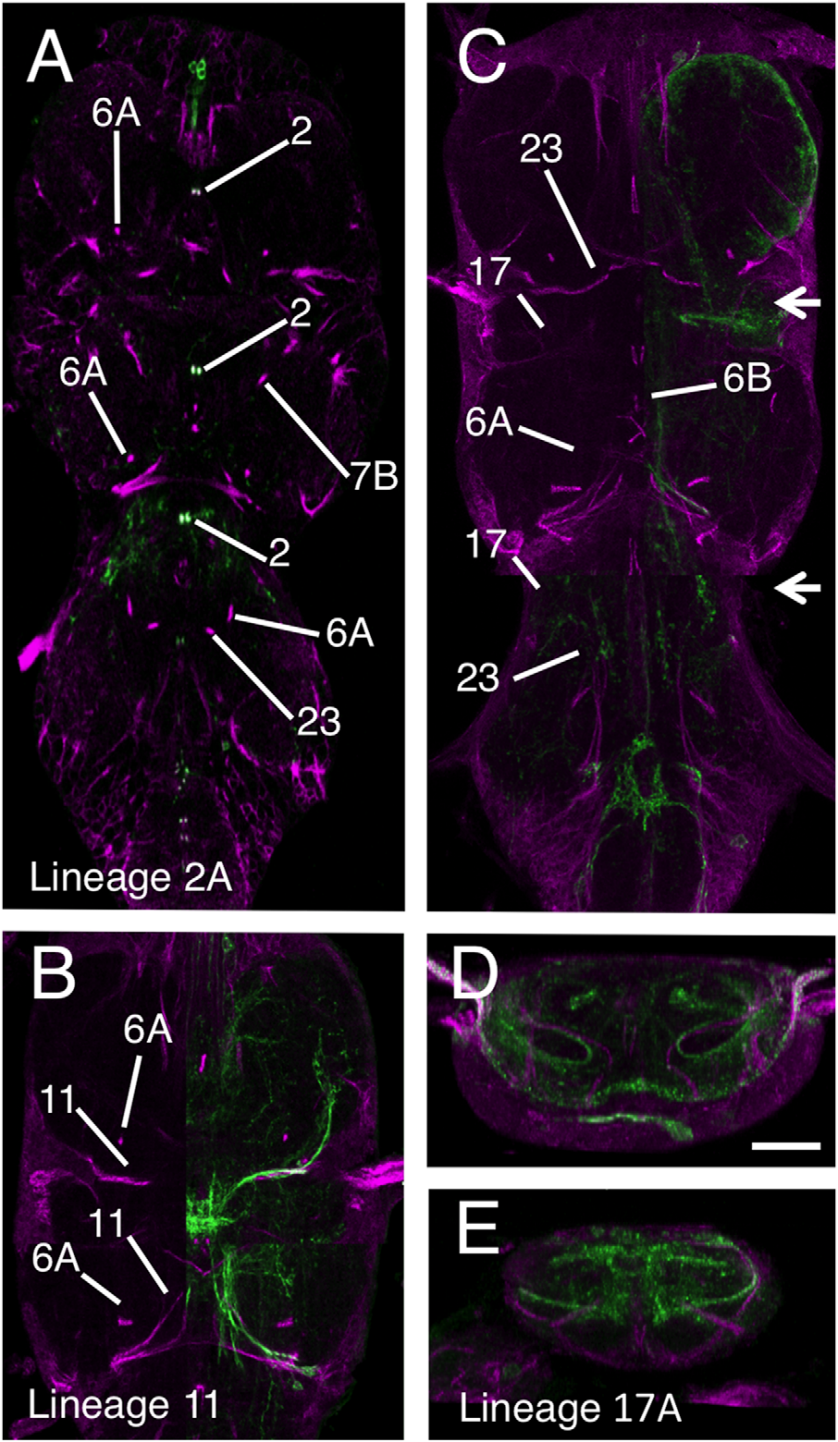
Validation of the hemilineage tract identification for the anterior medial lineages. Data organization as in Fig. 3. **(A)** Hemilineage 2A revealed by expression pattern of line R50G08. **(B)** Hemilineage 11 revealed by immortalization of the larval expression pattern of line R26B05. **(C–E)** Hemilineage 17A revealed by immortalization of the larval expression pattern of line R78A08. (D,E) Transverse sections through image shown in panel B at the levels indicated by the arrows. Scale bar = 100 μm.

The embryonic and postembryonic progeny of this NB can be readily visualized from larva to adult using the GAL4 line R50G08. Postembryonic extensions of the lineage are found only in the thoracic neuromeres and are represented by only the 2A hemilineage (Truman et al., 2010). The 2A tracts are readily identifiable at all stages, and show a consistent projection in all three thoracic neuromeres (Fig. 1A–C). In adult, 2A enters the neuropil from a mid‐ventral position close to the anterior midline and projects to the dorsal surface of the neuromere before turning laterally. Within each neuromere the two paired hemilineage 2A tracts form a characteristic doublet projection in the anterior half of the neuromere either side of the midline and are among the most easily recognized of the neuroglian bundles (Fig. 7A).

**Figure 8:**
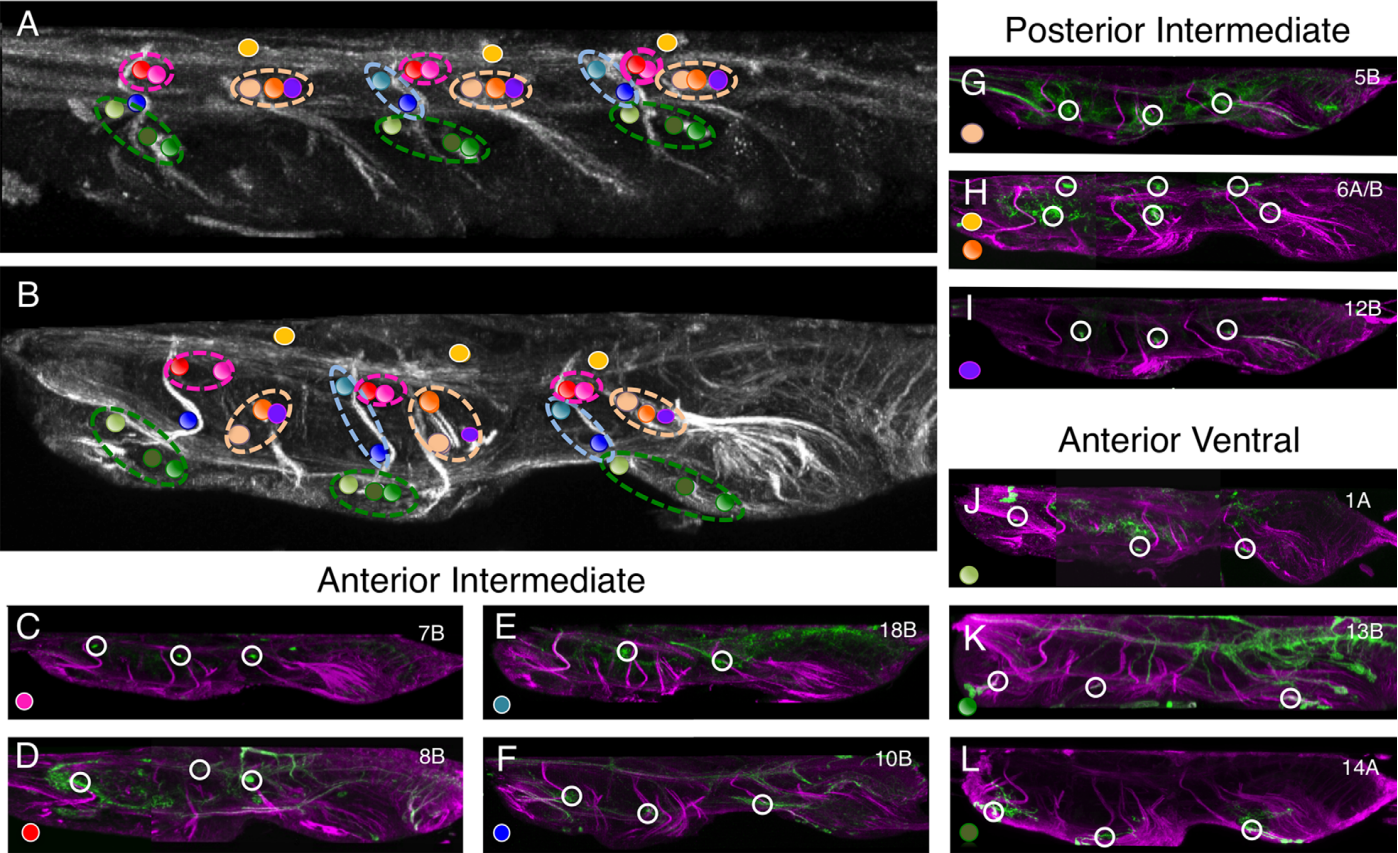
Identifying the larval commissures established by larval hemilineage in the adult VNS. **(A)** Sagittal section (10 μm) through the larval VNS labeled to reveal the neuroglian scaffold. **(B)** Sagittal section (10 μm) through the adult VNS labeled to reveal the neuroglian scaffold. The positions of the commissural processes of the postembryonic lineages are indicated with colored spots. The color of the spot indicates which lineage forms that commissure. The collective larval commissures are indicated by the dotted lines encircling the individual projections. **(C–L)** Sagittal sections (10 μm) through double‐labeled adult VNS showing the neuroglian scaffold (magenta) and the structure of the hemilineage revealed by GFP expression (green). (C–F) The lineages forming the larval anterior intermediate commissure. The lineages anterior to lineage 2 (10B and 18B) are in the blue circle and lineages posterior to lineage 2 (7B and 8B) are in the red circle. (G–I) The lineages forming the larval posterior intermediate commissure in the orange circle. (J–L) The lineages forming the larval anterior intermediate commissure in the green circle.

##### Lineage 0 (MNB)

Lineage 0 is produced by the median unpaired (NB0) and is present in all three thoracic neuromeres with a cell cluster located at the posterior midline of each neuromere. The 0 tract is readily identifiable in all three thoracic neuromeres at all stages of metamorphosis (Fig. 1A–C). The 0 tract originates as an unpaired tract from the cell cluster at the ventral posterior midline and projects anteriorly and dorsally along the midline between the 5B, 6B, and 12B tracts. In all three thoracic neuromeres, the 0 tract terminates on the midline in dorsal neuropil at the same plane as the 2A tract ( Figs. 1C5–6).

We were unable to find a GAL4 line that reliably preserves expression in lineage 0 to definitively identify the neuroglian tract but its characteristic features as an unpaired, midline bundle make its identification unequivocal from the neuroglian signal alone.

##### Lineage 11 (NB 6‐4 or 7‐2)

Lineage 11 is the most segmentally specialized lineage in the VNS, with a unique combination of hemilineages in each thoracic neuromere. 11A is present in T1 and T2, 11B is found only in T2 and both are absent from T3.

In the adult T1, 11A enters the VNS as part of medially projecting bundle that includes 11, 19, and 23 (Fig. 1C6) and cannot be resolved from these but is revealed by the immortalized expression of GFP in line R26B05, which shows that the lineage 11A neurites project posteriorly, but do not cross the midline (Fig. 7B).

In T2, 11A enters the neuropil as part of a tract that includes 19 (Figs. 1C4, 6). On entering the neuropil the tracts diverge and 11A projects anteromedially and anterior to 19, but stops short of the midline (Fig. 1C6). 11B projects more dorsally than 11A and enters the neuropil at a more dorsal point and makes a characteristic turn as it enters the neuropil to project anteriorly and terminate in the posterior lateral neuropil (Figs. 1C6, 7B).

##### Lineage 17 (NB 2‐5)

In larva lineage, 17 is one of three anterodorsal clusters. It is comprised of only the 17A hemilineage (Truman et al., 2010) and was found in only T2 and T3. It is characterized by a tract that projects medially towards the midline but turns sharply dorsally and hooks back upon itself to produce a characteristic hooked projection (Truman et al., 2004). In the adult, this shape is even more pronounced, forming a large loop in the anterior of the segment.

17A is seen at all stages (Fig. 1A5,B5) as a laterally originating tract that projects medially in the anterior third of the neuromere (Fig. 1A5,B5). In adult, 17A often stains weakly (Fig. 1C6) but in some examples it is clearly labeled. In both T2 and T3, 17A enters the neuropil from a dorsal lateral position in the anterior of the neuromere and projects anteromedially (Figs. 1C6, 7C–E). We had difficulty isolating a GAL4‐expressing line to confirm the identity of the 17A tract. The best genotype created by immortalization of line R78A08 marks 17A most of the time, but also reveals lineage 11, and occasionally other lineages. Despite this complication, the immortalized line showed that the adult 17A is still distinguished by the characteristic hook‐shaped projection (Fig. 7C–D).

## DISCUSSION

By defining the rules by which neuronal circuits are assembled during development, we have a chance to understand the functional logic and evolution of neuronal networks. As demonstrated with the vertebrate spinal cord, knowledge of the developmental origins of the interneuron pools has been vital to building an understanding of the functional organization of the complex sensory motor systems generating locomotor behavior (Grillner and Jessell, 2009). *Drosophila* is an equally powerful, well‐understood model for developmental biology, with amenable genetics and a sophisticated array of locomotor behaviors controlled by a relatively small nervous system. We have known for years that the neuronal populations controlling locomotion in the VNS are produced by distinct neural precursors called neuroblasts (Thomas et al., 1984). Truman et al. (2010) described the development and organization of the postembryonic secondary neurons in the larva and the modular nature of hemilineages. Despite the obvious power of a developmental approach and suitability of *Drosophila*, a development‐based analysis of circuitry in the VNS has been slow in coming. The reasons for this are due primarily to the complex changes that take place as the larval secondary neurons differentiate into their adult forms during metamorphosis and that the tools that allowed the analysis of the larval system, the neurotactin immunoreactivity, stopped at the onset of metamorphosis. Understanding how the developmental units in the larva integrate into the adult nervous system is very difficult without a tool to label the tracts.

Analyzing neuroglian expression at key stages in the metamorphosis of the adult VNS, we have traced the adult fate of each of the larval hemilineage neurite bundles to generate a map of neuroglian‐positive tracts in the adult VNS—based on the full set of 31 hemilineages that contribute interneurons to each hemisegmental unit of the VNS. By double labeling any neuron‐specific marker with neuroglian it is possible to determine precisely which hemilineage the neuron belongs to by identifying its associated neuroglian positive bundle. Thus, it is now possible to assign any secondary neuron in the thoracic neuromeres to its hemilineage of origin. This makes neuroglian expression a powerful tool in understanding the developmental organization of the adult VNS.

### Validation

Although tracing the neuroglian scaffold through metamorphosis was without major complications, it was nevertheless important that we independently validate the work. To do this we used the genetic toolkit developed by Harris et al. (2015) to preserve larval hemilineage‐specific GAL4 expression patterns into adult stage. Although the GFP signal in these immortalized lines was not always complete enough to describe the full projective field of the lineages, it was sufficient to confirm the identity of each tract. In some cases the signal was weak and only detectable at significant levels in the cell clusters and in the neuroglian bundles where their neurites are most tightly bundled. In others, although the larval expression was maintained in the complete hemilineage, the expression pattern does change and neurons not related to the original larval expression pattern are also revealed. In these cases care was taken to ensure that additional expression did not cause misidentifications. Despite these minor limitations, the immortalized expression was a powerful confirmation of the analysis of the neuroglian scaffold and we are confident that the identifications of the neuroglian tracts in all three thoracic neuromeres are correct.

### An anatomical framework for the VNS

The description of the scaffold of neuroglian tracts not only provides a tool to analyze the clonal origins of the VNS it also provides a powerful framework to help define its anatomical organization. An understanding of neuronal organization in the neuropil requires a frame of reference to define the structure of neurons and describe their anatomical relationships. The work of Merritt and Murphey (1992) and Boerner and Duch (2010) have provided such frameworks for the adult VNS based on the longitudinal tracts and commissures. The hemilineage tracts add another dimension to this architecture, revealing another set of organizational features within the neuropil.

One value of this framework is that it can be used to unravel the segmental organization of the fused neuropils of the VNS. Since each neuromere is founded by a specific set of NBs (Truman and Bate, 1988) and the neuroglian bundles produced by their progeny create a metamere‐specific set of anatomical markers that define each neuromere, we can use these structures to describe the neuromere boundaries. This is best seen in the horizontal plane of the mesothoracic neuromere (Fig. 1C). The mesothoracic neuropil is defined anteriorly by the tracts from the anterior mesothoracic lineages 2, 9, 10, 7, 8, 16, and 15, all of which project posteriorly into the mesothoracic neuromere. Similarly, the posterior margin of the mesothoracic neuromere is defined by the tracts from the posterior mesothoracic lineages 3, 6, 0, 21, 11, and 19, all of which project anteriorly into the mesothoracic neuromere. Thus, the mesothoracic neuromere is encompassed by the projections of the mesothoracic lineages. The same principle can be used to define the pro‐ and metathoracic neuropils (Fig. 1C).

From this analysis it is evident that there is a region of neuropil posterior to the lineage tracts of T1 and to the anterior lineage tracts of T2 that falls outside these boundaries. This region is the accessory mesothoracic neuropil (AMN), a morphologically distinct subdivision of the VNS (Merritt and Murphey, 1992) at the interface between the pro‐ and mesothoracic neuromeres. It is largely formed from the wing sensory afferents entering the VNS via the anterior dorsal mesothoracic nerve (Merritt and Murphey, 1992). Although the AMN is known and described as a distinct structure, the lineage tracts we present in this article provide a framework that can be used to define its structure and boundaries.

As well as defining the neuromere boundaries, the lineage tracts also define another nonsegmental feature of the VNS neuropil, the tectulum. Power (1948) defined the tectulum as a “distinct subdivision of the thoracic regions of the [VNS]. The region forms a saddle‐like structure located dorsally primarily over the mesothoracic neuropil but extending over the posteriormost region of the prothoracic neuromere and the anteriormost region of the metathoracic neuropil.” Despite subsequent work this remains the best definition of the tectulum. The neuroglian framework provides recognizable boundaries that circumscribes the tectulum (Fig. 2) and allows a more precise definition. The tectulum corresponds to the dorsal region of the neuropil posterior to the anteriormost limits of the 12B tracts in T1 but dorsal to the tracts from 12B, 6B, 23, 17, and 18 in T2. It extends posteriorly through T2 to the entry point of hemilineage 3A in T3. As with the neuromere structure, the neuroglian bundles provide a clear and precise tool for defining distinct regions of the VNS neuropil.

### Linking larval commissures to their adult counterparts

The neuroglian tracts and immortalized lineage‐specific GFP also reveal the developmental origins of adult VNS commissures. Truman et al. (2004) showed that the postembryonic lineages crossed the midline via specific and invariant commissural pathways and defined four larval commissures formed by central neurons. Using GFP to reveal the projections of the same neurons in the adult, we can now provide a definitive marker for each of these commissures and link the larval commissures to the adult and relate these to descriptions of the adult VNS commissures made by Power (1948; Fig. 8). Although the larval commissures are formed by the hemilineages are identifiable as distinct anatomical structures in larval stages, from our work it is evident that most of the larval commissures segregate as the different neuronal components are drawn apart by the expansion of the neuropil during pupation. As a consequence, the larval commissures are not immediately recognizable in the adult as gross anatomical structures in the adult VNS and can only be described with direct reference to the GFP expression in specific hemilineages as they cross the midline.

In the larva the 1A, 13B, and 14A tracts cross the midline in the anterior ventral commissure with 1A passing anterior to 2A and 13B and 14A passing posterior to 2A (Truman et al., 2004). In the adult these neurons relate to adult commissures, some of which were identified by Power, and others that were not. In T1 neither of the commissures formed by both 1A and 13B/14A was described by Power (1948). In T2, however, the 1A tract is the accessory prothoracic commissure and 13B/14A tract crosses in the ventral accessory commissure of the mesothoracic neuromere. In T3, the 1A tract forms the accessory commissure of the metathoracic neuromere but the 13B/14A commissure was not identified by Power (1948).

In larva, hemilineages 10 and 18 cross the midline in the anterior intermediate commissure. The adult projections from these hemilineages segregate to form two commissures, both of which pass anterior to 2A tract, with 10 being more ventral but neither correspond to a commissure described by Power (1948).

The tracts from hemilineages 5, 6B, 7, 8, and 12B form the posterior intermediate commissure in larva. In adult T2 and T3 neuromeres, the commissure formed by these neurons segregate into two commissures. The anteriormost contain hemilineages 7B and 8B and form the two most robust commissures of the adult VNS; in T2 they form the commissure of the mesothoracic neuromere and in T3 the haltere commissure. The commissure formed by 6B, 12B, and 5B, however, form a posterior commissure not identified by Power (1948).

The 6A tract is part of the posterior dorsal commissure in the larva, the dorsalmost of the larval commissures, and found in all three thoracic neuromeres. In adult, the commissure containing 6A is in the tectulum and forms part of three primary dorsal thoracic commissures: the posterior dorsal prothoracic commissure in T1, the posterior dorsal mesothoracic commissure in T2 and the posterior dorsal metathoracic commissure in T3.

In summary, the work presented in this article provides a key to understanding the developmental organization of the neuropil of the VNS, and enables work to understand clonal origin of different populations of neurons. With the simple expedient of colabeling neurons with anti‐neuroglian and identifying the neuroglian tract containing the primary neurites, it should be possible to identify parent lineage of virtually all secondary neurons in the VNS. From this knowledge it should be possible to place neurons into a functional and developmental context and begin to unravel the functional organization of neurons in the *Drosophila* VNS and other insect groups.

## CONFLICT OF INTEREST

The authors declare no conflicts of interest.

## CONTRIBUTIONS OF AUTHORS

All authors were fully involved in the production of data and had full access. All authors take responsibility for the integrity and accuracy of the data. DS was primarily responsible for the analysis of the metamorphosis of the neuroglian Scaffold. RH produced most of the immortalized larval expression data with some additional work done by DS. DWW provided assistance with the analysis of the neuroglian signal and identification of lineages. JWT provided support for all aspects of the work. DS drafted the article and produced all figures. JWT and DWW commented on and amended drafts.

**Table 1:** Antibodies Used

Antibody	Immunogen	Source	Dilution
Anti GFP	GFP isolated directly from the jellyfish *Aequorea victoria*	Molecular Probes (Invitrogen); Cat. no. A11122 RRID:AB_221569	1:500
Anti‐*Drosophila* Neuroglian	Nervous system‐specific 180 kD splice variant of *Drosophila* Neuroglian (Hortsch et al., 1990).	Developmental Studies Hybridoma Bank; Cat. no. BP 104 anti‐Neuroglian RRID:AB_528402	1:40

## Supporting information

This article includes Supplementary Material available via the Internet at http://www.interscience.wiley.com/jpages/xxxx-xxxx/suppmat.

Supporting Information Figure 1

Supporting Information Figure 2

Supporting Information Figure 3

Supporting Information Figure 4
